# Relationship between gray matter structure and age in children and adolescents with high-functioning autism spectrum disorder

**DOI:** 10.3389/fnhum.2022.1039590

**Published:** 2023-01-06

**Authors:** Fenfen Sun, Yue Chen, Yingwen Huang, Jing Yan, Yihong Chen

**Affiliations:** ^1^Center for Brain, Mind, and Education, Shaoxing University, Shaoxing, China; ^2^Department of Psychology, Shaoxing University, Shaoxing, China; ^3^Department of Otorhinolaryngology, The First People’s Hospital of Xiaoshan, Hangzhou, China

**Keywords:** autism spectrum disorder (ASD), gray matter structure, children and adolescents, age, regression analysis

## Abstract

**Objective:**

The present study used magnetic resonance imaging to investigate the difference in the relationship between gray matter structure and age in children and adolescents with autism spectrum disorder (ASD) and typically developing (TD) subjects.

**Methods:**

After screening T1 structural images from the Autism Brain Imaging Data Exchange (ABIDE) database, 111 children and adolescents (7–18 years old) with high-functioning ASD and 151 TD subjects matched for age, sex and full IQ were included in the current study. By using the voxel-based morphological analysis method, gray matter volume/density (GMV/GMD) maps were obtained for each participant. Then, a multiple regression analysis was performed for ASD and TD groups, respectively to estimate the relationship between GMV/GMD and age with gender, education, site, and IQ scores as covariates. Furthermore, a *z*-test was used to compare such relationship difference between the groups.

**Results:**

Results showed that compared with TD, the GMD of ASD showed stronger positive correlations with age in the prefrontal cortex, and a stronger negative correlation in the left inferior parietal lobule, and a weaker positive correlation in the right inferior parietal lobule. The GMV of ASD displayed stronger positive correlations with age in the prefrontal cortex and cerebellum.

**Conclusion:**

These findings may provide evidence to support that the brain structure abnormalities underlying ASD during childhood and adolescence may differ from each other.

## Introduction

Autism spectrum disorder (ASD) is a neurodevelopmental disorder that begins in early childhood, and its core symptoms are mainly impairments of social communication and language, repetitive stereotyped behaviors, and narrow interests, which seriously hinder the socialization process of child development (Baio et al., 2018). A previous survey showed that the prevalence of ASD was 1‰ for children aged 0–6 years, 1.53‰ for children aged 2–6 years, and approximately 2.59‰ for children aged 3–12 years (Rydzewska et al., 2019). Studies have shown that the prevalence of ASD in children under the age of 8 is 1/59 (Baio et al., 2018) and is 1/45 in children or adolescents aged 3–17 (Zablotsky et al., 2015), and the prevalence of adult autism is 9.8‰ (Brugha et al., 2016). Moreover, ASD patients in different age groups have different clinical symptoms. For example, a prior study has demonstrated that differences between verbal and non-verbal skills (especially visual processing) in children with ASD increase with age, and children with high non-verbal skills have greater impairments in social functioning (Joseph et al., 2002). Another study found that male children with ASD might exhibit more repetitive stereotyped behaviors after age 6 than before age 6 (Van Wijngaarden-Cremers et al., 2014). Additionally, adult ASD patients’ executive function and working memory worsen with age (Braden et al., 2017). Collectively, these findings suggest that the pathogenesis of ASD may be closely related to age.

In recent years, non-invasive magnetic resonance imaging (MRI) technology has been widely used in the study of the pathological mechanisms underlying diseases. Studies have shown that compared with typically developing (TD) subjects, the gray matter in brains of children with ASD aged 1 to 2 years showed overgrowth (Hazlett et al., 2005); the brain volume of those aged 2 to 4 years was significantly larger, but such a change was not found in the adult ASD population (Redcay and Courchesne, 2005). Moreover, this study also found that the gray matter volume changes of the frontotemporal cortex, limbic system and cerebellum in adult ASD patients were different from those of children and adolescents (Yang et al., 2016), and the cortical thickness decreased with age in children and adolescents with ASD but increased with age in adulthood (Van Rooij et al., 2018). These findings suggest that gray matter structure in the cortex of ASD patients shows age-specific alterations. As a widespread and lifelong developmental disorder, ASD seriously affects the quality of life of patients, especially children and adolescents with age ranged from 7 to 18 years (Dong et al., 2021; Zheng et al., 2021). However, so far, the relationship between gray matter structure and age in children and adolescents with high-functioning ASD and its difference with TD are still unclear.

The present study used MRI to calculate the gray matter volume/density (GMV/GMD) and compared the relationship between gray matter structure and age in ASD and TD individuals to explore the brain structure mechanism of the effect of age in ASD. We hope that our findings will provide a theoretical basis for behavioral intervention and educational countermeasures for children and adolescents with ASD.

## Materials and methods

### Participants and MRI data information

T1-weighted MRI data was downloaded from the Autism Brain Imaging Data Exchange (ABIDE^1^). All images were acquired with a 3T MRI scanner with a resolution of 1 × 1 × 1 mm^3^. More details of the scan sequence parameters can be found on the ABIDE website. Data collection at all sites was approved by the local Ethics Committee. The structural MRI images from a total of 1,081 subjects were downloaded at the initial stage. Then, the subjects were screened according to the following criteria: (1) right-handed; (2) full IQ ≥ 80; (3) 7 years old < age < 18 years old; (4) sample size ≥ 10 at each site (see Table 1 for site and sample size information); and (5) age, IQ, site origin, and gender showed no significant difference between the ASD and TD groups. The IQ of all subjects was measured by the Wechsler Abbreviated Scales of Intelligence (WASI), which includes the full intelligence quotient (FIQ), verbal intelligence quotient (VIQ) and performance intelligence quotient (PIQ) indicators. The symptoms of ASD patients were assessed by the Autism Diagnostic Interview Revised (ADI-R) scale. Finally, 111 ASD and 151 TD subjects entered the subsequent data analysis.

**TABLE 1:** Sites of participants where T1 images were obtained and corresponding sample sizes.

Site	ASD group	TD group
NYU	44	64
PITT	15	13
SDSU	13	19
STANFORD	14	15
UCLA	10	28
USM	15	12

NYU, New York University Langone Medical Center; PITT, University of Pittsburgh; SDSU, San Diego State University; STANFORD, Stanford University; UCLA, University of California, Los Angeles; USM, University of Utah; Yale, Yale Child Study Center.

## MRI data processing

Voxel-based morphological (VBM) analysis was performed for each subject by using the VBM toolkit within SPM.^2^ First, the T1 structural image was spatially registered to a MNI age-specific spatial template (Dong et al., 2020). Second, the registered image was segmented into white matter, gray matter, and cerebrospinal fluid by the built-in segmentation algorithm of the software, and further performed the bias correction of images to remove inhomogeneities in signal intensity. Finally, the bias-corrected gray matter image and the modulated gray matter image reflected density and volume, respectively. Both GMV and GMD maps were smoothed with a Gaussian smoothing kernel with a width at half maximum of 8 mm.

## Statistical analysis

This study used multiple regression analysis based on the SPM software module to assess the correlation between age and gray matter structure. This regression model was established with age as an independent variable, gray matter structure (GMV/GMD) as a dependent variable, and gender, education level, total GMV, site and three IQ scores as the covariates. All analyses were performed within a gray matter mask, with FDR correction (voxel-level *p* < 0.05, cluster size > 100 voxels) for multiple comparisons to reduce false-positive results in statistical analyses. After FDR correction for the multiple regression analysis in each group, we obtained two masks and then merged them into one, which included the brain regions showing significant correlations between GMD/GMV and age in ASD or TD or both groups. Next, we calculated the Pearson’s correlation between GMD/GMV and age for each region within the merged mask in ASD and TD groups, respectively. Finally, we performed a *z*-test to detect the difference of correlation coefficient between the two groups in each region.

## Results

### Demographic data

There were no significant differences in age, gender and IQ between the two groups (*p* > 0.05), as shown in Table 2.

**TABLE 2:** Comparison between groups of demographic data.

	ASD	TD	*t/*χ^2^	*P*-value
Age^a^	12.47 ± 3.01	12.87 ± 2.68	−1.13	0.26
Gender (male/female)^b^	98:13	122:29	2.67	0.10
FIQ^a^	109.02 ± 15.68	109.38 ± 12.62	−0.21	0.84
VIQ^a^	106.12 ± 16.22	108.84 ± 12.86	−1.52	0.14
PIQ^a^	109.94 ± 15.90	107.64 ± 13.93	1.24	0.23
ADI-R_social interaction	19.68 ± 5.22	–	–	–
ADI-R_verbal communication	15.82 ± 4.23	–	–	–
ADI-R_restricted and repetitive behavior	6.18 ± 2.34	–	–	–

^a^Represents independent-samples *t*-test; ^b^Represents chi-square test.

## Relationship between GMV/GMD and age

**FIGURE 1:**
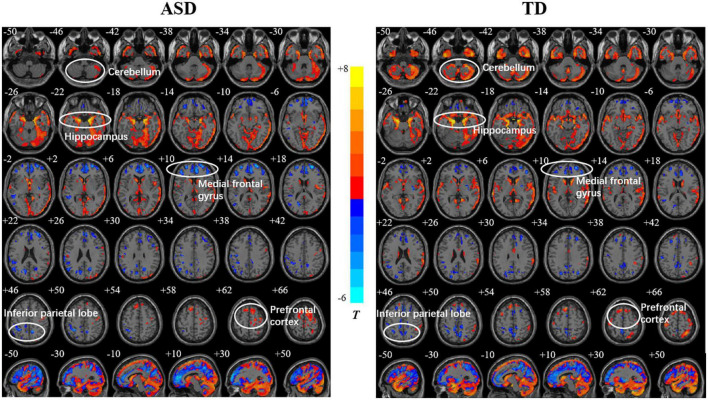
Relationships between age and gray matter density (GMD) in the autism spectrum disorder (ASD) and typically developing (TD) groups. The color bar shows the *T* value, in which warm and cold colors represent positive and negative correlations, respectively.

**FIGURE 2:**
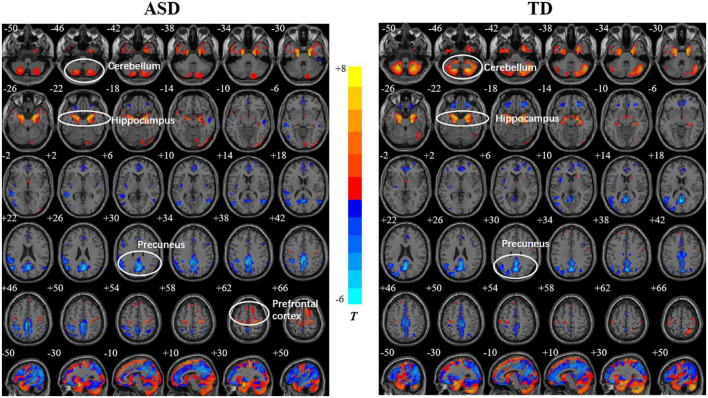
Relationships between age and gray matter volume (GMV) in the autism spectrum disorder (ASD) and typically developing (TD) groups. The color bar shows the *T* value, in which warm and cold colors represent positive correlation and negative correlations, respectively.

Both the ASD and TD groups showed positive correlations between age and the GMDs in the prefrontal cortex (PFC), orbitofrontal gyrus, occipital lobe, and hippocampus. The GMDs in the medial frontal lobe, anterior cingulate gyrus, and inferior parietal lobule (IPL) were negatively correlated with age in the two groups (Figure 1). However, the GMD in the cerebellum showed a positive correlation with age in the TD group but not in the ASD group. The two groups showed a positive correlation between GMV in the hippocampus as well as cerebellum and age and negative correlations between GMVs in the precuneus, posterior cingulate and IPL and age (Figure 2). However, a positive correlation between GMV in the prefrontal lobe and age was found only in the ASD group.

**FIGURE 3:**
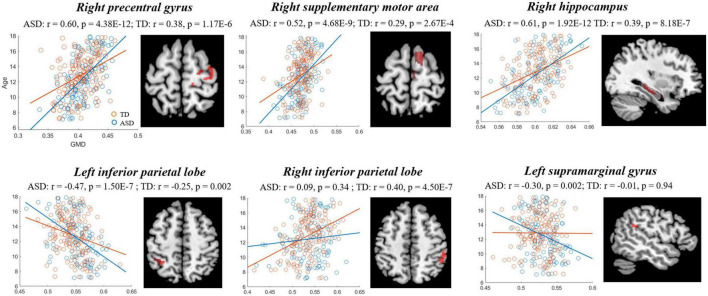
Correlations between gray matter density (GMD) and age showing significant differences between autism spectrum disorder (ASD) and typically developing (TD). Blue and red represent the ASD and TD, respectively.

**FIGURE 4:**
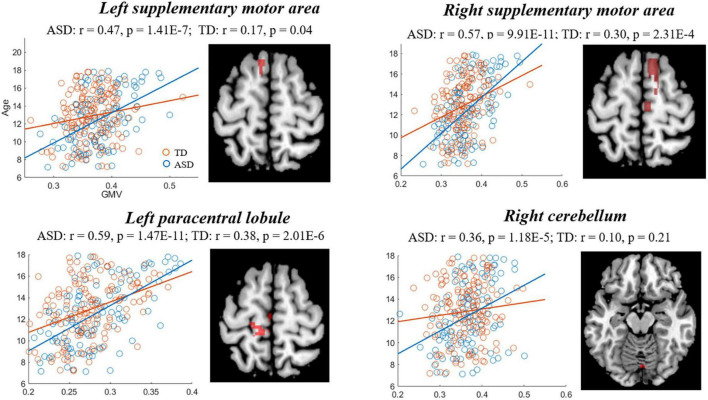
Correlations between gray matter volume (GMV) and age showing significant differences between autism spectrum disorder (ASD) and typically developing (TD). Blue and red represent the ASD and TD, respectively.

The comparisons between the groups found that compared with TD, the GMD of ASD showed stronger correlations with age in right PFC [precentral gyrus and supplementary motor area (SMA)], hippocampus, left IPL and supramarginal gyrus (SMG), and a weaker correlation in the right IPL (Figure 3, Table 3), while the GMV of ASD showed higher correlations with age in prefrontal cortex (bilateral SMA and left paracentral lobule), and right cerebellum (Figure 4, Table 3).

**TABLE 3:** Comparison of correlation between gray matter volume/density (GMV/GMD) and age.

Region	AAL	MNI			*z*-value	*P*-value
		*X*	*Y*	*Z*		
***GMD***						
Right precentral gyrus	2	47	−8	50	2.25	0.02
Right supplementary motor area	20	11	6	72	2.18	0.03
Right hippocampus	38	21	−5	−20	2.30	0.02
Left inferior parietal lobe	61	−32	−47	50	−2.08	0.04
Right inferior parietal lobe	62	41	−42	41	−2.59	0.01
Left supramarginal gyrus	63	−54	−44	26	−2.38	0.02
***GMV***						
Left supplementary motor area	19	−14	−3	72	2.73	0.006
Right supplementary motor area	20	11	8	63	2.66	0.008
Left paracentral lobule	69	−12	−27	65	2.18	0.03
Right cerebellum	112	6	−77	−14	2.13	0.03

## Discussion

This study investigated the relationship between gray matter structure and age in children and adolescents with high-functioning ASD. We found that the gray matter structure of the bilateral PFC, left IPL and cerebellum in the ASD showed stronger correlations with age, whereas that of the right IPL showed a weaker correlation with age in the ASD compared with TD. These findings suggest that the structural development of gray matter in high-functioning ASD patients is abnormal during childhood and adolescence, which may be the underlying structural brain mechanism of patients’ dysfunctions.

The PFC is one of the abnormal brain regions most frequently reported in ASD patients and is the core brain region of the neural mechanism of theory of mind (the ability to recognize and predict the mind of others). It plays a key role in executive control, cognitive development, and behavioral processing (Li et al., 2021). A previous study found that, compared with normal controls, the GMV of the PFC in ASD patients aged 6–16 years increased with age (DeRamus and Kana, 2015). In another study of ASD patients aged 7–29 years, Lin et al. (2015) found that GMV in the PFC increased with age in ASD patients but decreased with age in healthy controls. Foster et al. (2015) found that children with ASD (mean age 12 years) showed increased GMV in the PFC relative to normally developing children. Consistent with these results, the present study also found stronger positive correlations between both GMV and GMD in the PFC and age in the ASD group compared with the TD group. Our recent study revealed that the GMD and GMV in the PFC were respectively correlated with the verbal communication scores and restricted and repetitive behavior scores on the ADI-R in the ASD group (Sun et al., 2022). Therefore, the differences between ASD and the TD group with regard to the relationship between gray matter structure and age in the PFC may suggest that executive control and communication dysfunctions of ASD patients were related to the abnormal development of this brain region.

The IPL is involved in sensory input, especially visual and spatial localization (Bremmer et al., 2001). Cheng et al. (2011) found that the GMV in bilateral IPL decreased with the increase of age in autistic adolescents, while it increased with the elevation of age in TD individuals. Scheel et al. (2011) revealed that the left IPL of autistic individuals was thinner than that of the TD individuals and tended to decrease with age. Moreover, a recent ASD study found that the GMV of IPL in early childhood (0–5 years old) increased with age and then gradually decreased with age (5–20 years old) (Levman et al., 2018). Another study found that the GMV in the right IPL in the autism group (6–12 years old) were smaller than those in the TD group, and the GMV in the left IPL in the autism group (13–18 years old) was larger than that in the TD group. These findings suggest that the gray matter changes of bilateral IPL in autistic individuals are correlated with age, which could be related to the involvement of the lateralization of this brain structure (Wang et al., 2022). Similarly, the present study found that the GMD of left IPL showed a stronger negative correlation with age, whereas that of right showed a weaker positive correlation with age in ASD group than TD group. Moreover, prior studies have demonstrated that the left IPL of ASD individuals was associated with the formation of social pathological networks (Venkataraman et al., 2015) and the reduction of GMV in right IPL in ASD patients was positively correlated with the severity of social disorders (Cheng et al., 2011). Therefore, the abnormal change in gray matter structure in the IPL with age may be the structural mechanism underlying the abnormal functions in social interaction, although no significant correlation between GMV/GMD in the IPL and social interaction score on the ADI-R was found in ASD patients in our recent study (Sun et al., 2022).

Additionally, increasing evidence has demonstrated that, in addition to motor function, the cerebellum is also involved in higher functions such as executive control and social cognition and is involved in the pathological mechanism of ASD (Tsatsanis et al., 2003; D‘Angelo and Casali, 2013). A previous study found that GMV in the cerebellum was negatively correlated with age in adults with ASD, and the reduction in GMV in this region was positively correlated with the severity of autism symptoms (Osipowicz et al., 2015; Yang et al., 2016). Moreover, studies have shown that GMV in the cerebellum increases with age in ASD patients during childhood (Hazlett et al., 2005) and adolescence (Traut et al., 2018) and is positively correlated with cognitive performance (Moore et al., 2017). Similarly, the present study found a stronger positive correlation between GMV in the right cerebellum and age in the ASD than TD groups, suggesting a overgrowth of the gray matter structure of the cerebellum in children and adolescents with ASD.

There are several limitations in the present study. First, the MRI data used in this study was scanned from multiple sites, and thus, we could not correct for the effect of the differences in scanning parameters, although we added the site as a covariate in our regression model. Second, our study was a cross-sectional design, so we could not directly capture the relationships between GMD/GMV and age. In the future, a single-site study with a large sample size is needed to validate our findings, and an ongoing longitudinal study will explore the development of gray matter structures with age in children and adolescents with ASD. Finally, the gender difference in GMV/GMD in ASD patients needs to be explored in the future.

In summary, the present study found that gray matter structure in the frontoparietal regions and cerebellum showed different correlations with age between the ASD and TD groups, suggesting that the structural abnormalities in these regions may be related to the neurobiological mechanism of ASD.

## Data availability statement

Publicly available datasets were analyzed in this study. This data can be found here: Autism Brain Imaging Data Exchange (ABIDE, http://fcon_1000.projects.nitrc.org/indi/abide/).

## Ethics statement

The studies involving human participants were reviewed and approved by the Local Ethics Committee at all sites from the Autism Brain Imaging Data Exchange (ABIDE) database. Written informed consent to participate in this study was provided by the participants or their legal guardian/next of kin.

## Author contributions

FS designed and wrote the manuscript. YuC analyzed the data and wrote the manuscript. YH polished the language. JY collected the relevant literatures. YiC revised and improved the manuscript. All authors contributed to the article and approved the submitted version.

## Footnotes

1
http://fcon_1000.projects.nitrc.org/indi/abide/

2
https://www.fil.ion.ucl.ac.uk/~john/misc/VBMclass10.pdf
